# Myrothecium-like new species from turfgrasses and associated rhizosphere

**DOI:** 10.3897/mycokeys.51.31957

**Published:** 2019-04-18

**Authors:** Junmin Liang, Guangshuo Li, Shiyue Zhou, Meiqi Zhao, Lei Cai

**Affiliations:** 1 State Key Laboratory of Mycology, Institute of Microbiology, Chinese Academy of Sciences, Beichen West Road, Chaoyang District, Beijing 100101, China Institute of Microbiology, Chinese Academy of Sciences Beijing China; 2 College of Life Sciences, Hebei University, Baoding, Hebei Province, 071002, China Hebei University Baoding China; 3 College of Life Sciences, University of Chinese Academy of Sciences, Beijing 100049, China University of Chinese Academy of Sciences Beijing China; 4 College of Plant Protection, China Agricultural University, Beijing 100193, China Agricultural University Beijing China; 5 Forwardgroup Turf Service & Research Center, Wanning, Hainan Province, 571500, China Forwardgroup Turf Service & Research Center Wanning China

**Keywords:** Stachybotryaceae, soil fungi, turfgrass disease, multi-locus phylogeny, cup-shaped sporodochia

## Abstract

*Myrothecium* sensu lato includes a group of fungal saprophytes and weak pathogens with a worldwide distribution. *Myrothecium* s.l. includes 18 genera, such as *Myrothecium*, *Septomyrothecium*, *Myxospora*, all currently included in the family Stachybotryaceae. In this study, we identified 84 myrothecium-like strains isolated from turfgrasses and their rhizosphere. Five new species, i.e., *Alfariapoae*, *Alf.humicola*, *Dimorphisetaacuta*, *D.obtusa*, and *Paramyrotheciumsinense*, are described based on their morphological and phylogenetic distinctions. Phylogenies were inferred based on the analyses of sequences from four DNA loci (ITS, *cmdA*, *rpb2* and *tub2*). The generic concept of *Dimorphiseta* is broadened to include a third type of seta, i.e. thin-walled, straight with obtuse apices.

## Introduction

*Myrothecium* was first introduced by Tode (1790) based on *M.inundatum*. The typical characters of these fungi are cup-shaped sporodochia covered by a mass of slimy, green to black conidia. The generic concept of *Myrothecium* has been emended several times (Link 1809; von Höhnel 1905; Pidoplichko and Kirilenko 1971). Decock et al. (2008) reported that the genus *Myrothecium* is not monophyletic based on internal transcribed spacer regions and the intervening 5.8S rDNA (ITS). Chen et al. (2015) re-evaluated the phylogeny of *Myrothecium* based on ITS and elongation factor 1-alpha (EF1-α) gene sequences, suggesting the polyphyly of *Myrothecium* within Stachybotryaceae. These studies did not make taxonomic conclusions accordingly. Lombard et al. (2016) constructed a backbone tree of *Myrothecium* s.l. based on a multi-locus phylogeny and resolved *Myrothecium* s.l. to 18 genera including 13 new genera introduced. Under the current concept of *Myrothecium* sensu stricto, only two species were included, *M.inundatum* and *M.simplex* (Lombard et al. 2016).

Most myrothecium-like species are saprobes in soils (Ellis and Ellis 1985). Many species were named referring to their substrates such as *Alfariaterrestris*, *Albifimbriaterrestris*, *Simorphisetaterrestris* and *Parvotheciumterrestre*. Some species were also reported as weak plant pathogens. For instance, *Paramyrotheciumroridum* (syn. *Myrotheciumroridum*) can infect coffee plants, causing bark canker (Tulloch 1972). *Albifimbriaverrucaria* (syn. *Myrotheciumverrucaria*) is pathogenic to mulberry causing leaf spot (Murakami et al. 2005). In addition, myrothecium-like species are also well-studied for their natural compounds, which are able to inhibit the activity of liver cancer and tumors (Pope 1944; Okunowo et al. 2010). Some myrothecium-like species can also produce a cocktail of secondary metabolites, which have strong antifungal and antibiotic activity (Kobayashi et al. 2004; Liu et al. 2006; Ruma et al. 2015). Hereto, more than 50 of these bioactive compounds have been reported from *P.roridum* and *Alb.verrucaria* (Wagenaar and Clardy 2001).

In a survey of turfgrass diseases from 2017, a number of myrothecium-like strains were collected from leaves and roots of turfgrasses and their rhizosphere. The aim of this study was to characterize these strains based on morphology and molecular phylogenetic analyses.

## Materials and methods

### Fungal isolates

From May 2017 to March 2018, turfgrass diseases were investigated on cold-season species in Beijing and on warm-season species in Hainan Province. Atotal of 130 samples were collected. Each sample was treated as an underground part of soil sample and a ground part of diseased grasses. Soil samples were isolated following the modified dilution plate method (Zhang et al. 2017). Five grams of each soil sample were suspended in 30 mL sterile water in a 50 mL bioclean centrifuge tube. The suspension was mixed thoroughly using Vortex-Genie 2 (Scientific Industries, New York) with maximum speed and then diluted to a series of concentration, i.e., 10^-1^, 10^-2^, 10^-3^ and 10^-4^. The 100 μL suspensions of each concentration were spread on to antibiotic potato dextrose agar (PDA, 4 g potato starch, 5 g dextrose and 15 g agar, 50 mg ampicillin and streptomycin sulfate in 1 L sterile water). The first few samples suggested that 10^-2^ was the best-diluted concentration for colony pickup. Diseased samples were isolated following a tissue isolation protocol (Chen et al. 2015). All plates were incubated at room temperature (23–25 °C) for 3–4 weeks, and from which all single colonies were picked up and transferred to clean PDA plates. Purified strains were stored at 4 °C for further studies. For phylogenetic analysis, associated sequences of 73 myrothecium-like strains and one outgroup strain were retrieved from GenBank (NCBI, https://www.ncbi.nlm.nih.gov/; Table 1).

**Table 1.** Strains and NCBI GenBank accessions used in the phylogenetic analyses.

Species	Isolate no. ^a^	Host/Substrate	Country	NCBI accession numbers			
				*cmdA*	ITS	*tub2*	*rpb2*
* Myrothecium simplex *	CBS 582.93^T^	Decaying agaric	Japan	KU846439	NR145079	KU846537	–
	CBS 100287	* Russula nigricans *	Japan	KU846440	KU846457	KU846538	–
* M. inundatum *	CBS 275.48^T^ = IMI 158855	* Russula adusta *	England	KU846435	KU846452	KU846533	–
	CBS 116539	Agaric	Canada	KU846437	KU846454	KU846535	–
* Albifimbria lateralis *	CBS117712^T^	Unknown	USA	KU845865	KU845881	KU845957	KU845919
* Alb. terrestris *	CBS 1261^8^6T	Soil in mopane woodlands	Namibia	KU845867	KU845883	KU845959	KU845921
	CBS 109378 = NRRL 31066	Dead hardwood	USA	KU845866	KU845882	KU845958	KU845920
	CBS 127838	Soil	Namibia	KU845868	KU845884	KU845960	KU845922
	LC12196	rhizosphere soils of *Poa* sp.	China	MK500260	MK478879	MK500277	–
* Alb. verrucaria *	CBS 328.52^T^ = NRRL 2003 = ATCC 9095	deteriorated baled cotton	USA	KU845875	KU845893	KU845969	KU845931
	CBS 189.46 = IMI 140060	* Solanum tubersum *	Cyprus	KU845872	KU845889	KU845965	KU845927
	LC12191	Rhizosphere soils of *Poa* sp.	China	MK500255	MK478874	MK500272	MK500264
	LC12192	Rhizosphere soils of *Poa* sp.	China	MK500256	MK478875	MK500273	MK500265
	LC12193	Rhizosphere soils of *Poa* sp.	China	MK500257	MK478876	MK500274	MK500266
	LC12194	Rhizosphere soils of *Poa* sp.	China	MK500258	MK478877	MK500276	MK500267
	LC12195	Rhizosphere soils of *Poa* sp.	China	MK500259	MK478878	MK500275	MK500268
* Alb. viridis *	CBS 449.71^T^ = BCC 37540	Unknown	India	KU845879	KU845898	KU845974	KU845936
	CBS 127346	Soil	USA	KU845880	KU845899	KU845975	KU845937
* Alfaria. ossiformis *	CBS 324.^5^4T	Prairie soil	USA	KU845977	KU845984	KU846015	KU846002
***Alf.humicola* sp. nov.**	CGMCC3.19213^T^ = LC12143	Rhizosphere soils of *Poa* sp.	Beijing, China	MH885432	MH793291	MH793317	MH818829
	LC12144	Rhizosphere soils of *Poa* sp.	Beijing, China	MH885434	MH793293	MH793318	MH818830
***Alf.poae* sp. nov.**	CGMCC3.19198^T^ = LC12140	Leaves of *Poa* sp.	Hainan, China	MH885419	MH793278	MH793314	MH818826
	LC12141	Rhizosphere soils of *Poa* sp.	Hainan, China	MH885420	MH793279	MH793315	MH818828
	LC12142	Rhizosphere soils of *Poa* sp.	Hainan, China	MH885421	MH793280	MH793316	MH818827
* Alf. putrefolia *	CBS 112037^T^	Rotten leaf	Brazil	–	KU845985	KU846016	KU846003
	CBS 112038	Rotten leaf	Brazil	–	KU845986	KU846017	KU846004
* Alf. terrestris *	CBS 477.91^T^	Soil	Turkey	KU845979	KU845988	KU846019	KU846006
* Alf. thymi *	CBS 447.83^T^	* Thymus serpyllum *	The Netherlands	KU845981	KU845990	KU846021	–
* Capitofimbria compacta *	CBS 111739^T^	Decaying leaf	Brazil	KU846261	KU846287	KU846404	KU846349
	MUCL 50238	Bark	Zimbabwe	–	KU878556	KU878559	KU878558
						
* Dimorphiseta terrestris *	CBS 127345^T^	Soil collected in *tallgrass prairie*	USA	KU846284	KU846314	KU846431	KU846375
***D.acuta* sp. nov.**	CGMCC3.19208^T^ = LC12122	Rhizosphere soils of *Poapratensis*	Beijing, China	MH885429	MH793288	–	MH818815
	LC12123	Leaves of *Digitariasanguinalis*	Beijing, China	MH885417	MH793276	MH793300	MH818811
	LC12124	Leaves of *Poapratensis*	Beijing, China	MH885418	MH793277	MH793297	MH818812
***D.acuta* sp. nov.**	LC12125	Rhizosphere soils of *Poapratensis*	Beijing, China	MH885427	MH793286	MH793298	MH818813
	LC12126	Rhizosphere soils of *Poapratensis*	Beijing, China	MH885428	MH793287	MH793299	MH818814
	LC12127	Rhizosphere soils of *Poapratensis*	Beijing, China	MH885430	MH793289	MH793301	MH818820
***D.obtusa* sp. nov.**	CGMCC3.19206^T^ = LC12128	* Poa pratensis *	Beijing, China	MH885426	MH793285	MH793307	MH818816
	LC12129	Rhizosphere soils of *Agrostisstolonifera*	Beijing, China	MH885415	MH793274	MH793303	MH818821
	LC12130	Rhizosphere soils of *Poapratensis*	Beijing, China	MH885431	MH793290	MH793308	MH818817
	LC12131	rhizosphere soils of *Poa* sp.	Beijing, China	MH885416	MH793275	MH793304	–
	LC12132	Rhizosphere soils of *Festucaarundinacea*	Beijing, China	MH885422	MH793281	MH793305	MH818818
	LC12133	Rhizosphere soils of *Poapratensis*	Beijing, China	MH885423	MH793282	MH793306	MH818819
	LC12134	Roots of *Poapratensis*	Beijing, China	MH885424	MH793283	MH793309	–
	LC12135	Roots of *Poapratensis*	Beijing, China	MH885425	MH793284	MH793302	–
* Gregatothecium humicola *	CBS 205.96^T^	Soil	Papua New Guinea	KU846285	KU846315	KU846432	KU846376
* Peethambara sundara *	CBS 646.77^T^	Dead twig	India	–	KU846471	KU846551	KU846509
	CBS 521.96 = MUCL 39093	Dead twig	Nepal	–	KU846470	KU846550	KU846508
* Inaequalispora prestonii *	CBS 175.73^T^	Forest soil	Malaysia	KU846286	KU846316	KU846433	KU846377
	MUCL 52636	rhizoplane and roots of plants	Ecuador	–	KY389317	KY366447	KY389355
* Myxospora masonii *	CBS 174.73^T^	Leaves of *Glyceria* sp.	England	KU846445	KU846462	KU846543	KU846500
* My. graminicola *	CBS 116538^T^	Decaying grass leaf	USA	KU846444	KU846461	KU846542	KU846499
* My. aptrootii *	CBS 101263^T^	Leaf litter	China	KU846441	KU846458	KU846539	KU846496
* My. musae *	CBS 265.71^T^	*Musa* sp.	Madagascar	–	KU846463	KU846544	KU846501
	CPC 25150	Tarspot lesion	South Africa	KU846446	KU846464	KU846545	KU846502
* My. crassiseta *	CBS 731.^8^3T	Dead twig	Japan	KU846442	KU846459	KU846540	KU846497
	CBS 121141 = NRRL 45891	Pyrenomycete	Hawaii	KU846443	KU846460	KU846541	KU846498
* Paramyrothecium humicola *	CBS 127295^T^	Soil collected in tallgrass prairie	USA	–	KU846295	KU846412	KU846356
* P. parvum *	CBS 257.35^T^	*Viola* sp.	United Kingdom	–	KU846298	KU846415	KU846359
	CBS 142.422= IMI 155923= MUCL 7582	Dune sand	France	KU846268	KU846297	KU846414	KU846358
* P. foeniculicola *	CBS 331.51^T^	*Foeniculumvulgare* leaf sheath	The Netherlands	–	KU846292	KU846409	KU846354
* P. nigrum *	CBS 116537^T^	Soil	Spain	KU846267	KU846296	KU846413	KU846357
	LC12188	Rhizosphere soils of *Poa* sp.	China	MK500252	MK478871	MK500269	MK500261
* P. cupuliforme *	CBS 1277^8^9T	Surface soil in desert	Namibia	KU846264	KU846291	KU846408	KU846353
* P. viridisporum *	CBS 873.85^T^	Soil	Turkey	KU846278	KU846308	KU846425	KU846369
	CBS 125835	Soil	USA	KU846280	KU846310	KU846427	KU846371
* P. acadiense *	CBS 123.96^T^	* Tussilago farfara *	Canada	–	KU846288	KU846405	KU846350
* P. terrestris *	CBS 564.86^T^	Soil	Turkey	KU846273	KU846303	KU846420	KU846364
	CBS 566.86	Soil	Turkey	KU846275	KU846305	KU846422	KU846366
* P. tellicola *	CBS 478.91^T^	Soil	Turkey	KU846272	KU846302	KU846419	KU846363
* P. foliicola *	CBS 113121^T^	Decaying leaf	Brazil	KU846266	KU846294	KU846411	–
	CBS 419.93	Air	Cuba	KU846265	KU846293	KU846410	KU846355
* P. breviseta *	CBS 544.75^T^	Unknown	India	KU846262	KU846289	KU846406	KU846351
* P. roridum *	CBS 357.89^T^	*Gardenia* sp.	Italy	KU846270	KU846300	KU846417	KU846361
	CBS 212.95	Water	The Netherlands	KU846269	KU846299	KU846416	KU846360
	CBS 372.50 = IMI 140050	*Coffea* sp.	Colombia	KU846271	KU846301	KU846418	KU846362
* P. guiyangense *	GUCC 201608S01^T^	*Soil*	Guiyang, China	KY196193	KY126418	KY196201	–
	HGUP 2016-8001	*Soil*	Guiyang, China	KY196192	KY126417	KY196200	–
* P. verruridum *							
	HGUP 2016-8006^T^	*Soil*	Guizhou, China	KY196197	KY126422	KY196205	–
***P.sinense* sp. nov.**	CGMCC3.19212^T^ = LC12136	Rhizosphere soils of *Poa* sp.	Beijing, China	MH885437	MH793296	MH793313	MH818824
	LC12137	Rhizosphere soils of *Poa* sp.	Beijing, China	MH885436	MH793295	MH793312	MH818822
	LC12138	Rhizosphere soils of *Poa* sp.	Beijing, China	MH885433	MH793292	MH793310	MH818823
	LC12139	Rhizosphere soils of *Poa* sp.	Beijing, China	MH885435	MH793294	MH793311	MH818825
* Parvothecium terrestre *	CBS 198.89^T^	Soil in virgin forest	Brazil	KU846449	KU846468	KU846548	KU846506
* Neomyrothecium humicola *	CBS 310.96^T^	Soil	Papua New Guinea	KU846448	KU846467	–	KU846505
* Gregatothecium humicola *	CBS 205.96^T^	Soi	Papua New Guinea	KU846285	KU846315	KU846432	KU846376
* Xepicula crassiseta *	CBS 392.71^T^	Soil	Spain	KU847222	KU847247	KU847337	KU847296
* X. jollymannii *	CBS 276.48^T^= MUCL 11830	* Nicotiana tabacum *	Malawi	KU847223	KU847248	KU847338	KU847297
	CBS 126168	Soil	Namibia	KU847224	KU847250	KU847340	KU847298
* X. leucotricha *	CBS 131.64= IMI 103664= ATCC 16686	Soil	India	KU847225	KU847251	KU847341	KU847299
	CBS 483.78	Soil	Colombia	KU847228	KU847254	KU847344	KU847302
* Smaragdiniseta bisetosa *	CBS 459.82^T^	Rotten bark	India	KU847206	KU847229	KU847319	KU847281
* Striaticonidium brachysporum *	CBS 513.71 ^T^ = IMI 115293	Dune sand	Iran	KU847209	KU847232	KU847322	KU847284
* S. brachysporum *	CBS 131.71= IMI 158441= ATCC 22270	Soil	Ukrain	KU847207	KU847230	KU847320	KU847282
	LC12189	Rhizosphere soils of *Poa* sp.	Beijing, China	MK500253	MK478872	MK500270	MK500262
	LC12190	Rhizosphere soils of *Poa* sp.	Beijing, China	MK500254	MK478873	MK500271	MK500263
* S. synnematum *	CBS 479.85^T^	Palm leaf	Japan	KU847218	KU847242	KU847332	KU847292
* S. cinctum *	CBS 932.69^T^	Soil	The Netherlands	KU847216	KU847239	KU847329	KU847290
	CBS 277.48 = IMI 001526	Soil	New Zealand	KU847213	KU847236	KU847326	KU847288
* S. humicola *	CBS 388.97	Soil	Papua New Guinea	KU847217	KU847241	KU847331	KU847291
* Tangerinosporium thalictricola *	CBS 317.61^T^ = IMI 034815	* Thalictrum flavum *	UK	KU847219	KU847243	KU847333	–
* Xenomyrothecium tongaense *	CBS 598.80^T^	*Halimeda* sp.	Tonga	KU847221	KU847246	KU847336	KU847295
* Virgatospora echinofibrosa *	CBS 110115	* Theobroma cacao *	Ecuador	KU847220	KU847244	KU847334	KU847293
	MUCL 39092 = ATCC 200437	* Trewia nudiflora *	Nepal	–	KU847245	KU847335	KU847294
* Fusarium sambucinum *	CBS 146.95	* Solanum tuberosum *	UK	KM231391	KM231813	KM232078	KM232381

† ATCC: American Type Culture Collection, Manassas, USA; BCC: BIOTEC Culture Collection, National Center for Genetic Engineering and Biotechnology (BIOTEC), Bangkok, Thailand; CBS: CBS-KNAW Fungal Diversity Centre, Utrecht, The Netherlands; CGMCC: China General Microbiological Culture Collection Center, Beijing, China; GUCC: Guizhou University Culture Collection, Guiyang, China; HGUP: Herbarium of the Department of Plant Pathology, Guizhou University, China; IMI: International Mycological Institute, England, UK; LC: Collection of Lei Cai, Institute of Microbiology, Chinese Academy of Sciences, Beijing, China; MUCL: Mycothèque de l’Université Catholique de Louvian, Belgium; NRRL: Northern Regional Research Laboratory, USA. ^T^ Ex-type and ex-epitype cultures.

### Morphology and culture characteristics

Descriptions of macromorphological features are based on 7-d old materials incubated in the dark at room temperature (20–25 °C) and grown on potato dextrose agar (2% w/w; PDA), oatmeal agar (OA), cornmeal agar (CMA) and synthetic low-nutrient agar (SNA; Nirenberg 1981). Color description followed the color guide by Kornerup and Wanscher (1978). Digital images of colonies were made with a Nikon Eclipse 80i light microscope (Tokyo, Japan) with differential interference contrast (DIC) illumination and a LV2000 digital camera (Beijing, China). Slides mounted in clear lactic acid were also prepared to observe conidiogenesis, conidiophores and conidia.

### DNA extraction and PCR amplification

Genomic DNA was extracted from 1–2 weeks’ old cultures grown on potato dextrose agar (2% w/w; PDA) incubated at room temperature using a modified Cetyltrimethyl Ammonium Bromide (CTAB) method (Rogers and Bendich 1994). Partial sequences of four genes, ITS, RNA polymerase II second largest subunit (*rpb2*), *β-tubulin* (*tub2*) and calmodulin (*cmdA*) gene sequences were amplified using the following pairs of primers, ITS1 and ITS4 (White et al. 1990) for ITS, RPB2-5F2 and RPB2-7cR (O’Donnell et al. 2007) for *rpb2*, Bt2a and Bt2b (Glass and Donaldson 1995) for *tub2* and CAL-228F (Carbone and Kohn 1999) and CAL2Rd (Groenewald et al. 2013) for *cmdA.* Amplification for each locus followed the PCR protocols as described in Lombard et al. (2016). The PCR was performed in a 25 μL reaction volume including 2.5 μL 10 × PCR Buffer (Dingguo, Beijing, China), 2 mM MgCl_2_, 50 μM dNTPs, 0.1 μM of each primer, 0.5 U Taq DNA polymerase and 10 ng genomic DNA. PCR reactions were conducted in ProFlex^TM^ PCR system (Applied Biosystems, California, USA) under the following reaction conditions: predenaturation at 94 °C for 5 min, followed by 35 cycles of denaturation at 94 °C for 30 s, annealing at 52 °C (for ITS) or 54 °C (for *rpb2* and *cmdA*) or 56 °C (for *tub2*) for 40 s and elongation at 72 °C for 1 min, a final elongation at 72 °C for 5 min.

The purified PCR products were sequenced in both forward and reverse directions on an ABI-3730 XL DNA Analyzer (Applied Biosystems, California, USA). The sequences were checked and manually corrected where necessary. A consensus contig was assembled with BioEdit v. 7.0.9 (Hall 1999) and the reference sequences were downloaded from GenBank (Table 1). Sequences were aligned with MAFFT v. 7 (Kazutaka and Standley 2013) and manually trimmed to equal length by cutting the unaligned sequences at both ends.

### Phylogenetic analyses

Phylogenetic analyses were based on Bayesian inference (BI) and Maximum Likelihood (ML). For BI analysis, the optimal evolutionary model was estimated in MrModeltest v. 2.3 (Nylander 2004) using the Akaike Information Criterion (AIC) for each locus. For the selected substitution models for each locus see Table 2. MrBayes v. 3.2.1 (Ronquist and Huelsenbeck 2003) was used to generate tree topology and a Markov Chain Monte Carlo (MCMC) algorithm of four chains was started with a random seed and a burn in of first 25% trees. The MCMC analysis lasted until the average standard deviation of split frequencies came below 0.01. The ML analysis was performed using RAxML servers (http://phylobench.vital-it.ch/raxml-bb/index.php), with a maximum likelihood bootstrap (LB) of 1,000 replicates, under the GTR-GAMMA model (Stamatakis 2006).

## Results

**Figure 1.**
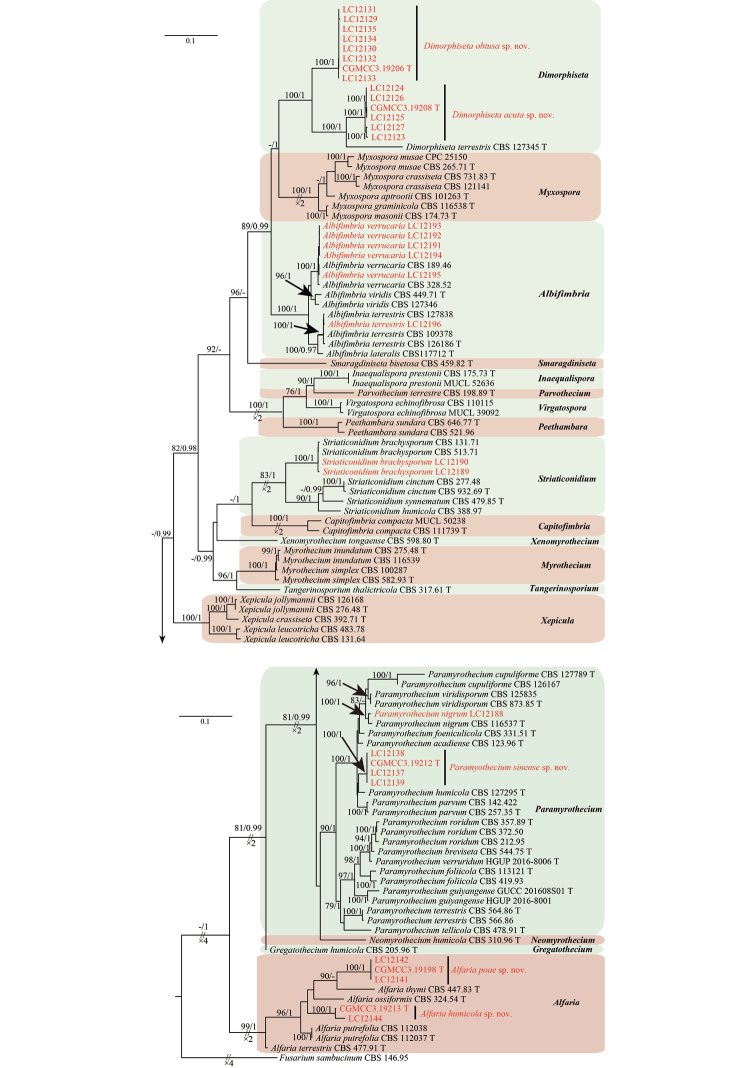
The ML consensus tree inferred from a four-locus concatenated alignment (ITS, *cmdA*, *rpb2* and *tub2*). Bootstrap values (1,000 replicates) over 70% for ML and posterior probability (PP) over 0.95 are added to the left of a node (ML/PP). The type strains are labeled with “T”. Strains obtained from this study are in red. The tree is rooted using *Fusariumsambucinum* (CBS 146.95).

In this study, 603 fungal strains were isolated. Based on colony morphologies and preliminary sequence comparison of ITS via BLASTn in GenBank, 84 myrothecium-like strains were selected. Phylogenetic analyses of above 84 strains were performed on single locus and concatenated datasets (ITS, *cmdA*, *tub2* and *rpb2*), with 70 strains in *Myrothecium* s.l. as reference and *Fusariumsambucinum* (CBS 146.95) as outgroup. After alignment, the concatenated datasets of four loci contained 569 characters (with gaps) for ITS, 318 for *tub2*, 732 for *cmdA* and 724 for *rpb2*. The characters of different alignments and statistics of phylogenetic analyses were shown in Table 2. The four single locus trees of all strains showed essentially similar topology (Supp. materials [Supplementary-material S1]1–4), with only minor differences affecting unsupported nodes on the trees. The resulting multi-locus ML tree was presented in Fig. 1 together with BI posterior probability values. Among 84 myrothecium-like strains, 14 strains were identified as four known species, *Albifimbriaverrucaria* (10 strains), *Alb.terrestris* (1 strain), *Striaticonidiumbrachysporum* (2 strains) and *Paramyrotheciumnigrum* (1 strain). The rest of them were grouped into five distinct clades with high supported values. Based on the morphological and phylogenetic distinctions, five novel species (i.e. *Alfariahumicola*, *Alf.poae*, *Dimorphisetaacuta*, *D.obtusa* and *Paramyrotheciumsinense*) were described in this paper.

**Table 2.** Characteristics of the different datasets and statistics of phylogenetic analyses used in this study.

Locus†	Number of sites*				Evolutionary model‡	Number of tree sampled in B	Maximum-likelihood statistics	
	Total	Conserved	Phylogenetically informative	B unique patterns			Best tree optimised likelihood	Tree length
ITS	569	334	193	247	GTR+I+G	7501	-32666.73	5.36
*tub2*	318	168	140	159	HKY+I+G			
*cmdA*	732	258	381	490	HKY+I+G			
*rpb2*	724	360	367	367	GTR+I+G			

† ITS, the internal transcribed spacer regions and 5.8s rRNA gene; *tub2*, *β-tubulin*; *cmdA*, calmodulin; *rpb2*: RNA polymerase II second largest subunit. * B = Bayesian inference. ‡ G: Gamma distributed rate variation among sites. GTR: Generalised time-reverisble. I: Proportion of invariable sites. HKY: Hasegawa-Kishino-Yano.

### Taxonomy

#### Dimorphiseta

Taxon classificationFungiHypocrealesStachybotryaceae

L. Lombard & Crous., Persoonia. 36: 188. 2016. emend. J.M.Liang & L.Cai.

Dimorphiseta terrestris L. Lombard & Crous. Persoonia. 36: 188. 2016. (Type species)

##### Note.

*Dimorphiseta* was a monotypic genus, introduced based on *D.terrestris*, which showed both type I (thin-walled, flexuous to circinate, narrowing to a sharp apex) and type II (thick-walled, straight to slightly curved, narrowing to a sharp apex) setae. Our study demonstrated that there is a third type of setae (type III: thin-walled, straight, terminating in an obtuse apex) in the genus.

#### Dimorphiseta acuta

Taxon classificationFungiHypocrealesStachybotryaceae

J.M. Liang, G.S. Li & L. Cai sp. nov.

829693

Fig. 2

##### Type.

China, Beijing, isolated from rhizosphere soils of *Poapratensis*, 26 Aug 2017, J.M. Liang, holotype HMAS 247957, dried culture on PDA, ex-holotype culture CGMCC3.19208 = LC12122.

##### Description.

*Colonies* on PDA, CMA and OA approx. 7–8 cm diam. after 7 d at room temperature (approx. 25 °C), mycelium white and abundant, with conidiophores forming on the aerial mycelium, carrying slimy olivaceous green to black conidial masses, reverse on PDA buff. *Conidiomata* sporodochial, stromatic, superficial, cupulate to discoid, scattered, rarely gregarious, irregular in outline, 50–300 μm diam., 60–150 μm deep, consisting of bundles of parallel, longitudinal, closely compacted hyphae, terminating in whorls of 3–5 conidiogenous cells, covered by an olivaceous green to black slimy mass of conidia without marginal hyphae. *Stroma* poorly developed, hyaline, of a textura angularis. *Setae* arising from the conidial mass, thick-walled, subhyaline, smooth, 5–15-septate, tapering to sharp apices, 120–370 μm long, 10–13 μm wide at the broadest part, 2–4 μm wide at the apex. *Conidiophores* macronematous, irregularly, unbranched, smooth to lightly verrucose, arising from the basal stroma. *Conidiogenous cells* phialidic, subcylindrical, hyaline, smooth, 10–20 μm long, 2–3 μm wide. *Conidia* aseptate, smooth, hyaline, ellipsoidal, rounded at the base, pointed at the apex with a funnel-shaped appendage, 7–12 × 2–3 μm (av. 10 ± 0.7 × 3 ± 1.3 μm, n = 50).

**Figure 2.**
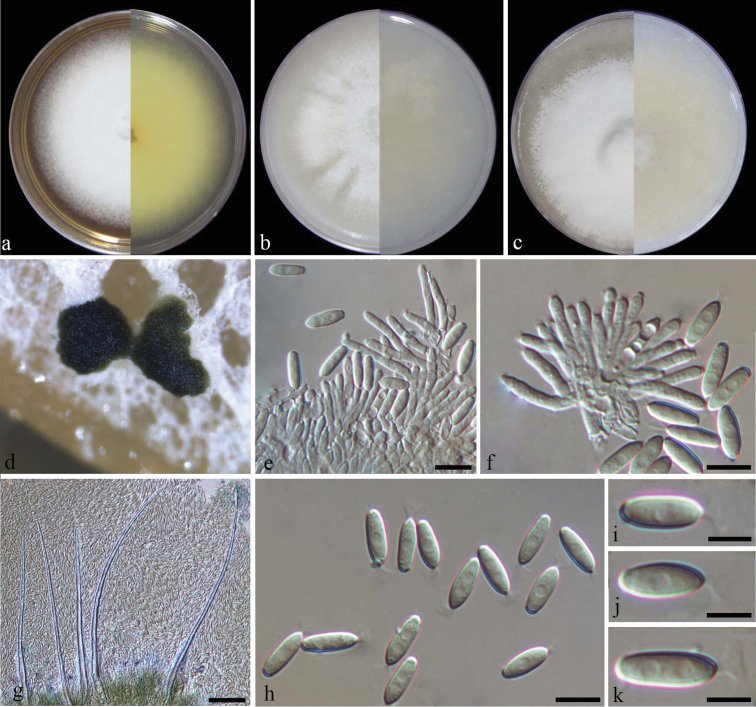
*Dimorphisetaacuta* (from ex-type strain CGMCC3.19208) **a–c** colony on PDA, CMA, OA**d** conidiomata on SNA**e** conidiophores **f** conidiogenous cells **g** setae **h–k** conidia. Scale bars: 5 μm (**e, f, h**): 50 μm (**g**); 2 μm (**i, j, k**).

##### Distribution.

China.

##### Etymology.

Name refers to the setae with tapered and sharp apices.

##### Additional isolates examined.

China, Beijing, from leaves of *Digitariasanguinalis*, 21 Aug 2017, J.M. Liang, LC12123; China, Beijing, from leaves of *Poapratensis*, 21 Aug 2017, J.M. Liang, LC12124; China, Beijing, from rhizosphere soils of *P.pratensis*, 21 Aug 2017, J.M. Liang & G.S. Li, LC12125, 21 Jul 2017, J.M. Liang, LC12126, 25 Jul 2017, J.M. Liang, LC12127.

##### Notes.

The multi-locus phylogenetic analyses indicated that *D.acuta* formed a sister clade to *D.terrestris*, but differs from the latter in the type and size of setae. *Dimorphisetaterrestris* produces both types of setae, the thin-walled and circinate type (Type I) and the thick-walled sharp-edged type (Type II), whereas *D.acuta* only produces the type I setae. In addition, the setae of *D.acuta* are much longer and wider than that in *D.terrestris* (120–370 μm × 10–13 μm vs. 70–95 × 3–4 μm) (Lombard et al. 2016). Morphologically, *D.acuta* should also be compared with *M.miconiae* and *M.xigazense*, which also produce sharp-edged setae. *Myrotheciummiconiae*, however, differs from *D.acuta* in producing 1-septate conidia (Alves et al. 2010), while *M.xigazense* differs in producing conidia that are truncate at both ends (Wu et al. 2014).

#### Dimorphiseta obtusa

Taxon classificationFungiHypocrealesStachybotryaceae

J.M. Liang, G.S. Li & L. Cai sp. nov.

829694

Fig. 3

##### Type.

China, Beijing, isolated from rhizosphere soils of *P.pratensis*, 23 Jun 2017, J.M. Liang, holotype HMAS 247954, ex-holotype culture CGMCC3.19206 = LC12128.

##### Description.

*Colonies* on PDA, OA and CMA approx. 5–6 cm diam. after 7 d at room temperature (approx. 25 °C), mycelium white and abundant, with conidiophores forming on the aerial mycelium, carrying slimy olivaceous green to black conidial masses, reverse on PDA pale luteous to buff. *Conidiomata* sporodochial, stromatic, superficial, scattered, rarely gregarious, oval to elongate or irregular in outline, 60–280 µm diam., 40–120 µm deep, with a setose fringe surrounding green to black slimy mass of conidia. *Stroma* poorly developed, hyaline, smooth to verrucose, of textura angularis. *Setae* arising from the basal stroma, thin-walled, 3–6-septate, unbranched, hyaline, smooth, 80–250 µm long, 2–4 µm wide at the broadest, terminating in a blunt apex. *Conidiophores* macronematous, irregularly, unbranched, smooth to lightly verrucose, arising from the basal stroma, up to 18 μm long. *Conidiogenous cells* phialidic, hyaline, smooth to verrucose, cylindrical, 7–19 × 2–3 μm, becoming narrowed at the tip with collarette. *Conidia* aseptate, ellipsoidal or cylindrical, hyaline, smooth, rounded both ends, with a funnel-shaped apical appendage, 9–11 × 2–4 μm (av. 10 ± 0.5 × 3 ± 0.3 μm, n = 50).

**Figure 3.**
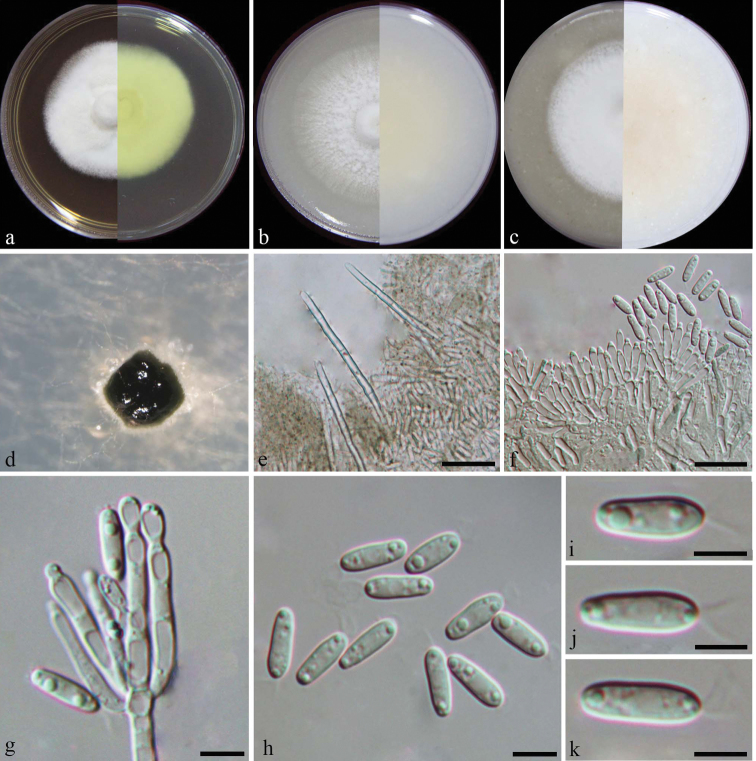
*Dimorphisetaobtusa* (from ex-type strain CGMCC3.19206) **a–c** colony on PDA, CMA, OA**d** conidioma on SNA**e** setae **f** conidiophores **g** conidiogenous cells **h–k** conidia. Scale bars: 50 μm (**e**); 10 μm (**f, g**); 5 μm (**h**); 2 μm (**i, j, k**).

##### Distribution.

China.

##### Etymology.

Named refers the setae with obtuse apices.

##### Additional isolates examined.

China, Beijing, from rhizosphere soils of *Agrostisstolonifera*, 24 Jul 2017, J.M. Liang, LC12129; China, Beijing, from rhizosphere soils of *P.pratensis*, 25 Aug 2017, J.M. Liang & G.S. Li, LC12130, 19 Jul 2017, J.M. Liang, LC12133; China, Beijing, from rhizosphere soils of *Poa* sp., 19 Jul 2017, J.M. Liang, LC12131; China, Beijing, from rhizosphere soils of *Festucaarundinacea*, 19 Jul 2017, J.M. Liang, LC12132; China, Beijing, from leaves of *P.pratensis*, 23 Jun 2017, J.M. Liang, LC12134, LC12135.

##### Notes.

*Dimorphisetaobtusa* formed a highly supported cluster with *D.terrestris* and *D.acuta*, but can be distinguished from the latter two by having setae with erect and obtuse apices. In addition, *D.obtusa* is also morphologically similar to two old un-sequenced *Myrothecium* taxa, i.e. *M.biforme* and *M.dimorphum*, but both of these two taxa have two types of conidia. *Myrotheciumbiforme* produces short cylindrical and ellipsoidal to navicular conidia (Jiang et al. 2014) and *M.dimorphum* has ovate and ellipsoidal conidia (Watanabe et al. 2003).

#### Alfaria humicola

Taxon classificationFungiHypocrealesStachybotryaceae

J.M. Liang, G.S. Li & L. Cai sp. nov.

829696

Fig. 4

##### Type.

China, Beijing, Olympic Park, from rhizosphere soil of *Poa* sp., 13 Dec 2017, S.Y. Zhou, holotype HMAS 247955, ex-holotype culture CGMCC3.19213 = LC12143.

##### Description.

*Colonies* on PDA, CMA and OA approx. 7–8 cm diam. after 7 d at 25 °C. *Hyphae* hyaline, smooth, branched, 1–2 μm wide. *Conidiomata* sporodochial, stromatic, superficial, cupulate to discoid, scattered to gregarious, oval to elongate or irregular in outline, 50–200 μm diam., 70–150 μm deep, without setose hyphae, covered by a green to black agglutinated slimy mass of conidia. *Stroma* well-developed, hyaline, of textura globulose or textura angularis. *Setae* absent. *Conidiophores* arising from the basal stroma, unbranched or branched, initially hyaline and smooth, becoming pigmented and verrucose with age, 11–25 µm long. *Conidiogenous cells* phialidic, cylindrical to allantoid, initially hyaline and smooth becoming pigmented and verrucose with age, 14–33 × 2–3 µm. *Conidia* aseptate, smooth, hyaline, elongated ellipsoidal to limoniform, straight, 7–9(–10) × 2–3 µm (av. 8 ± 0.6 × 3 ± 0.2 µm, n = 50).

**Figure 4.**
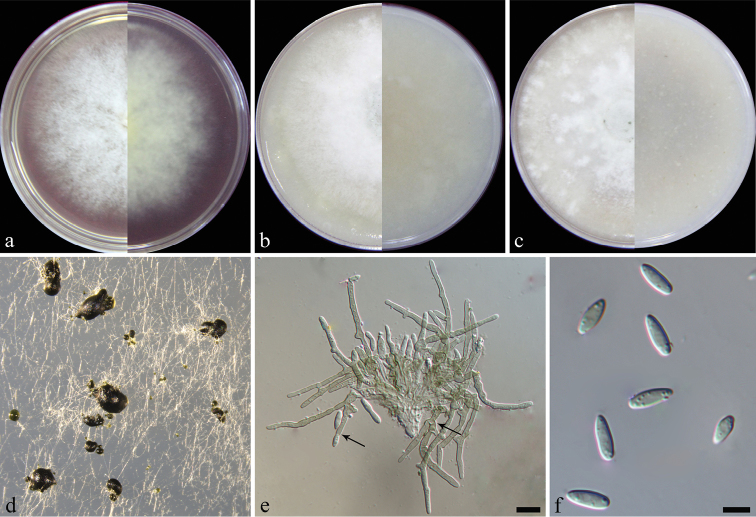
*Alfariahumicola* (from ex-type CGMCC3.19213) **a–c** colony on PDA, CMA, OA**d** conidiomata on SNA**e** sporodochial conidioma, arrows showing branched conidiosphores and conidiogenous cells **f** conidia. Scale bars: 10 µm (**e**); 5 µm (**f**).

##### Distribution.

China.

##### Etymology.

Name refers the substrate, soil, from which this fungus was isolated.

##### Additional isolate examined.

China, Beijing, Olympic Park, from rhizosphere soil of *Poa* sp., 13 Dec 2017, S.Y. Zhou, LC12144.

##### Notes.

*Alfariahumicola* represents another distinct lineage in *Alfaria* (Fig. 1). *Alfariahumicola* lacks setae, distinguishing it from *Alf.caricicola* and *Alf.thymi*. Furthermore, the conidiogenous cells of *Alf.humicola* (14–33 × 2–3 µm) are much longer than that of *Alf.arenosa* (5–10 × 1–2 µm), *Alf.ossiformis* (5–10 × 2–3 µm) and *Alf.terrestris* (5–11 × 1–3 µm). Compared with those old *Myrothecium* taxa lacking sequences, *Alf.humicola* is morphologically similar to *M.atrocarreum* (Berkeley & Broome, 1877), *M.conicum* (Fuckel, 1870), *M.ellipsosporum* (Fuckel, 1866), *M.fragosianum* (Saccardo, 1917), *M.leucomelas* (Höhnel, 1925) and *M.oryza* (Saccardo, 1917), but *Alf.humicola* produces limoniform conidia which makes it distinguishable. In addition, the conidiogenous cells of *Alf.humicola* show conspicuous collarettes which were not described in previous old taxa.

#### Alfaria poae

Taxon classificationFungiHypocrealesStachybotryaceae

J.M. Liang, G.S. Li & L. Cai sp. nov.

829697

Fig. 5

##### Type.

China, Hainan Province, Haikou, isolated from leaves of *Imperatacylindrica*, 10 Mar 2018, J.M. Liang and L. Cai, holotype HMAS 247953, ex-holotype culture CGMCC3.19198 = LC12140.

##### Description.

*Colonies* on PDA, CMA and OA with white aerial mycelium, approx. 6–7 cm diam. after 7 d at 25 °C, giving rise to dark green or blank sporodochia scattered or gregarious on the surface, covered by olivaceous green pillars of conidia, reverse on PDA sienna. *Hyphae* hyaline, smooth, branched, 1–2 μm wide. *Conidiomata* synnematous, solitary, 60–250 μm high, 30–80 μm wide at the base, 60–150 μm at the apex, with setose hyphae surrounding a green agglutinated mass of conidia. *Stroma* well developed, hyaline, of textura angularis. *Setae* absent. *Conidiophores* arising from the basal stroma, branched, initially hyaline and becoming pigmented and verrucose with age covered by an olivaceous green mucoid layer, up to 30 µm long. *Conidiogenous cell* phialidic, clavate to cylindrical, hyaline, smooth, 5–10 × 1–2 µm, becoming pigmented and verrucose with age, with conspicuous collarettes and periclinal thickenings. *Conidia* aseptate, smooth, hyaline, ellipsoidal to fusiform, 6–8 × 2–3 µm (av. 7 ± 0.4× 2 ± 0.2 µm, n = 50).

**Figure 5.**
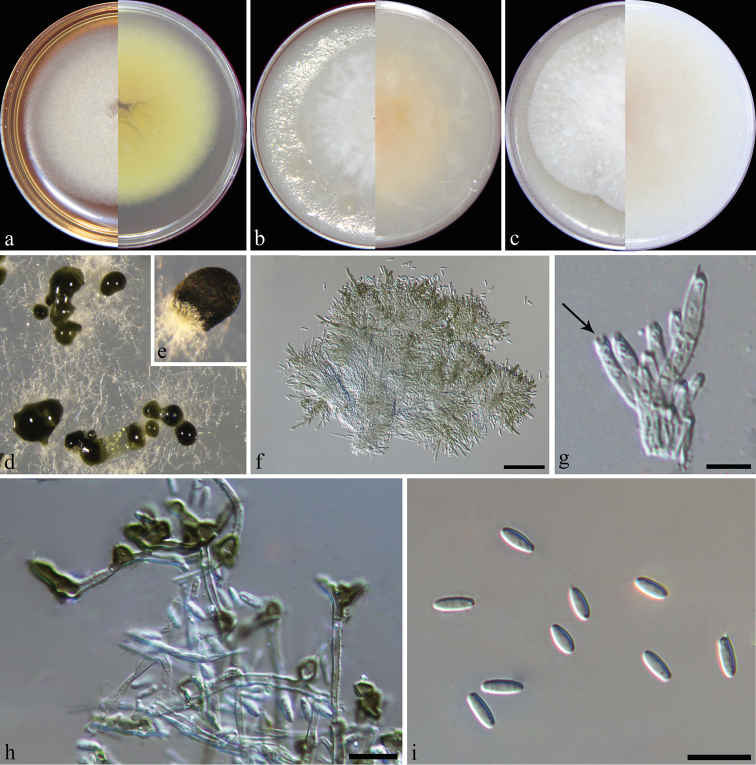
*Alfariapoae* (from ex-type strain CGMCC3.19198) **a–c** colony on PDA, CMA, OA**d–e** conidiomata on SNA**f** synnematous conidioma **g** conidiogenous cells, the arrow showing conspicuous collarette **h** aged conidiophores **i** conidia. Scale bars: 50 μm (**f**); 5 μm (**g**); 10 μm (**h, i**).

##### Distribution.

China.

##### Etymology.

Name refers the host, *Poa* sp., from which this fungus was isolated.

##### Additional isolate examined.

China, Hainan, from leaves of *Imperatacylindrica*, 10 Mar 2018, J.M. Liang & Lei Cai, LC12141, LC12142.

##### Notes.

*Alfariapoae* formed a well-supported clade in *Alfaria* (Fig. 1). Similar to *Alf.ossiformis* and *Alf.terrestris*, *Alf.poae* does not produce setae surrounding the sporodochia, distinguishing it from *Alf.caricicola* and *Alf.thymi*. *Alfariapoae* produces ellipsoidal to fusiform conidia, which are different from the ossiform conidia produced by *Alf.ossiformis* (Lombard et al. 2016). The conidia of *Alf.terrestris* have basal hilum which was not observed in *Alf.poae*. In addition, *Alf.poae* shares morphological characters with several un-sequenced *Myrothecium* taxa, such as *M.atrocarneum* (Berkeley & Broom, 1877), *M.conicum* (Fuckel, 1870), *M.ellipsosporum* (Fuckel, 1866) and *M.leucomelas* (Höhnel, 1925). Because the descriptions of *M.atrocarneum*, *M.conicum* and *M.ellipsosporum* were not elaborate enough, these old species are not distinct from *Alf.poae* yet. Future comparisons should be made when these old species are epitypified by fresh collections. Although *M.leucomelas* (host: *Sumbaviaerotttleroidis*; location: Bulacan, Luzon) had a detailed description, it cannot be epitypified by *Alf.Poae*, because *Alf.poae* was collected from a distinct location and plant host. Taking the above special characters into account, we considered introducing a new species, *Alfariapoae*.

#### Paramyrothecium sinense

Taxon classificationFungiHypocrealesStachybotryaceae

J.M. Liang, G.S. Li & L. Cai sp. nov.

829698

Fig. 6

##### Type.

China, Beijing, Olympic Park, from rhizosphere soil of *Poa* sp., 13 Dec 2017, S.Y. Zhou, holotype HMAS 247956, ex-holotype culture CGMCC3.19212 = LC12136.

##### Description.

*Colonies* on PDA, CMA and OA approx. 5–6 cm diam. after 7 d at 25 °C. *Hyphae* white, hyaline, smooth, branched, 1–2 μm wide, reverse on PDA pale luteous. *Conidiomata* sporodochial, stromatic, cupulate, superficial, scattered or gregarious, oval or irregular in outline, 80–600 μm diam., 50–150 μm deep, with a white setose fringe surrounding an olivaceous green to black agglutinated slimy mass of conidia. *Stroma* poorly developed, hyaline, of textura angularis. *Setae* arising from stroma, thin-walled, hyaline, 1–3-septate, straight to flexuous, 45–90 μm long, 1–3 μm wide, tapering to an acutely rounded apex. *Conidiophores* arising from the basal stroma, consisting of a stipe and a penicillately branched conidiogenous apparatus; stipes unbranched, hyaline, septate, smooth, 20–30 × 2–3 μm; primary branches aseptate, unbranched, smooth, 13–40 × 2–3 μm; secondary branches aseptate, unbranched, smooth, 8–15 × 2–3 μm; terminating in a whorl of 3–6 conidiogenous cells; conidiogenous cell phialidic, cylindrical to subcylindrical, hyaline, smooth, straight to slightly curved, 7–16 × 1–3 μm, with conspicuous collarettes and periclinal thickenings. *Conidia* aseptate, hyaline, smooth, cylindrical, 6–7 × 2–3 μm (av. 7 ± 0.3 × 2 ± 0.2 μm, n = 40), rounded at both ends.

**Figure 6.**
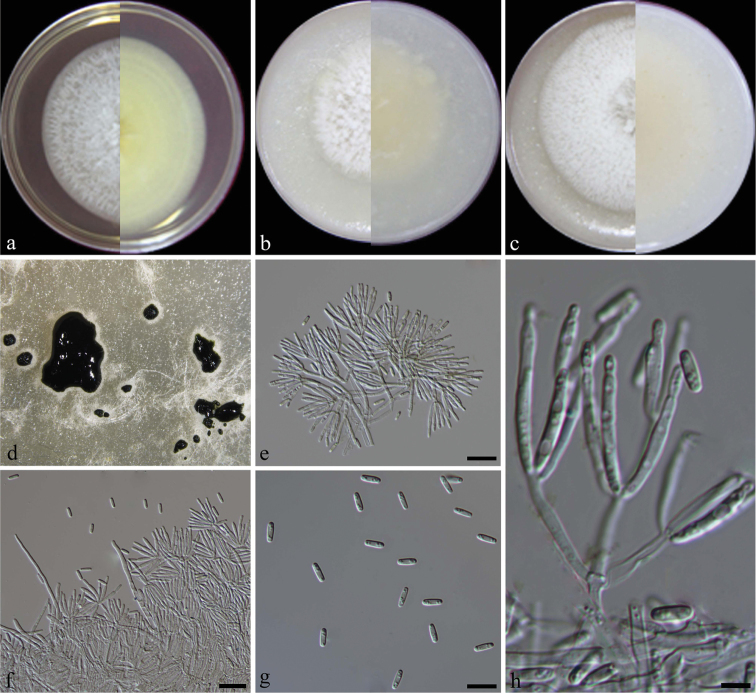
*Paramyrotheciumsinense* (from ex-type CGMCC3.19212) **a–c** colony on PDA, CMA, OA**d** conidiomata on SNA**e** sporodochial conidioma **f** setae **g** conidia **h** conidiogenous cells. Scale bars: 20 μm (**e, f**) ; 10 μm (**g**); 5 μm (**h**).

##### Distribution.

China.

##### Etymology.

Named after the country of collection, China.

##### Additional isolate examined.

China, Beijing, Olympic Park, from rhizosphere soils of *Poa* sp., 13 Dec 2017, S.Y. Zhou, LC12137, LC12138, LC12139.

##### Notes.

Lombard et al. (2016) introduced a new genus, *Paramyrothecium*, based on an epitype of *Myrotheciumroridum* Tode, 1790. Gams (2016) pointed out that *Myrotheciellacatenuligera*, the type species of *Myrotheciella* was listed as a synonym of *P.roridum* by Lombard et al. (2016), thus *Paramyrothecium* is illegitimate and *Myrotheciella* should be the correct name for *Paramyrothecium*. However, the original description of *Myrotheciellacatenuligera* suggested that it lacks seta (Spegazzini 1911), thus is clearly different from the morphological circumscription of *P.roridum*. Therefore, we do not agree with the treatment of Lombard et al. (2016) of listing *Myrotheciellacatenuligera* as a synonym of *P.roridum*.

*Paramyrotheciumsinense* formed a highly supported distinct clade closely related to *P.humicola*. The setae of this species are terminated with obtuse apices, dissimilar to the acute apices in *P.humicola*. In addition, the conidiophore stipes (20–30 μm long) and primary branches (13–40 μm long) of *P.sinense* are much longer than those of *P.humicola* (stipe, 12–22 μm long; primary branches, 7–17 μm long) (Lombard et al. 2016). Among old un-sequenced taxa in *Myrothecium*, only *M.biforme* and *M.dimorphum* show seta with obtuse apices, but both taxa produce two types of conidia (Jiang et al. 2014; Watanabe et al. 2003).

## Discussion

The ITS has been shown to be insufficient to delineate the myrothecium-like species. With the additions of partial sequences of *rpb2*, *cmdA* and *tub2*, phylogenetic relationships within Stachybotryaceae could be better resolved (Lombard et al. 2016). In this study, we isolated fungi from rhizosphere soils, leaves and roots of several turfgrasses, and our phylogenetic analyses based on concatenated four loci together with the morphological characters supported the recognition of five novel species in Stachybotryaceae.

By comparing the topologies of the four single-locus trees, incomplete lineage sorting was discovered in *Dimorphiseta*. Based on the single-locus trees of ITS and *rpb2*, *D.acuta*, *D.obtusa* and *D.terrestris* grouped together (Supp. materials 1, 4). Whereas in the single-locus phylogenetic analyses based on *tub2* and *cmdA*, *D.obtusa* grouped distantly from *D.acuta* and *D.terrestris*, but close to *Myxospora* and *Albifimbria* species (Supp. materials 2, 3). Three *Dimorphiseta* species are similar in the conidial shape and size (7–19 μm long), which are distinct from the shorter conidia in *Albifimbria* (4–8 μm long) and *Myxospora* (4–6 μm long) species (Tulloch 1972; Lombard et al. 2016). Conidia with a funnel-shaped apical appendage are a distinct feature of three *Dimorphiseta* species, but they are absent in all *Myxospora* species and most *Albifimbria* species (Lombard et al. 2016). Furthermore, the *rpb2* and 28S ribosomal DNA combined dataset, which was suggested to delimit generic boundaries of myrothecium-like species (Lombard et al. 2016) revealed that the three *Dimorphiseta* species clustered together (Supp. material 6: Table S1, Supp. material 5).

In the multi-locus sequence analysis of *Myrothecium* s.l. by Lombard et al. (2016), thirteen new genera were introduced including several monotypic genera, such as *Dimorphiseta*, *Capitofimbria*, *Gregatothecium* and *Neomyrothecium*. In this study, we reported two new species in *Dimorphiseta* (*D.acuta* and *D.obtusa*). With this addition, the generic concept of *Dimorphiseta* is slightly expanded for including a third type of setae. Hereto, *Dimorphiseta* is the genus with the most variable types of seta among *Myrothecium* s.l., which might be useful in the generic delimitation in *Myrothecium* s.l. (Lombard et al. 2016).

Lombard et al. (2016) narrowed the concept of *Myrothecium* s.s. to only include species with sporodochia or mononematous conidiophores producing conidia shorter than 5 μm in green slimy masses without mucoid appendages. Whether or not a conidial size should be defined in the generic concept remained debatable. Because many *Myrothecium* published recently produced much longer conidia, e.g. *M.chiangmaiense* (4–7 μm) (Dai et al. 2017), *M.uttaraditense* (10–15 μm) (Dai et al. 2017), *M.thailandicum* (6.5–10 μm) (Dai et al. 2017), *M.septentrionale* (8.5–12 μm) (Tibpromma et al. 2017), *M.variabile* (12.5–16.5 μm) (Wu et al. 2014) and *M.xigazense* (2.5–15 μm) (Wu et al. 2014). These above species were identified, either based on morphology only or with a single molecular locus (ITS), and should be better confirmed for their generic placement when more data are available. Currently, there are 90 records of *Myrothecium* in Index Fungorum (Jan 10, 2019), and 25 names have been successively transferred to other genera, i.e., *Capitofimbria*, *Melanconis*, *Striaticonidium*, *Xepicula* (Lombard et al. 2016), *Digitiseta* (Gordillo and Decock 2018). Only a limited number of the remaining species in *Myrothecium* have available molecular data (Dai et al. 2017; Tibpromma et al. 2017), as most of these taxa have no living cultures. We agree with Gams (2016) that these unvisited taxa are still important when the original descriptions are sufficiently clear to recognize a species. They should be epitypified in future studies when fresh collections with living cultures are available, and before that, descriptions of new taxa in this group should be made carefully with the inclusion of these un-sequenced taxa in morphological comparisons.

## Supplementary Material

XML Treatment for Dimorphiseta

XML Treatment for Dimorphiseta acuta

XML Treatment for Dimorphiseta obtusa

XML Treatment for Alfaria humicola

XML Treatment for Alfaria poae

XML Treatment for Paramyrothecium sinense

## Supplementary materials

Supplementary material 1 Figure S1. The ML consensus tree inferred based on *ITS* partial sequence with bootstrap values for ML (> 70%) and posterior probability (PP) (PP > 0.95) labeled to the left of a node (ML/PP) Data type: phylogenetic data Explanation note: The type strains were labeled with “T”. Strains obtained from this study are in red. This dataset is made available under the Open Database License (http://opendatacommons.org/licenses/odbl/1.0/). The Open Database License (ODbL) is a license agreement intended to allow users to freely share, modify, and use this Dataset while maintaining this same freedom for others, provided that the original source and author(s) are credited.Junmin Liang, Guangshuo Li, Shiyue Zhou, Meiqi Zhao, Lei Cai

Supplementary material 2 Figure S2. The ML consensus tree inferred based on *tub2* partial sequence with bootstrap values for ML (> 70%) and posterior probability (PP) (PP > 0.95) labeled to the left of a node (ML/PP) Data type: phylogenetic data Explanation note: The type strains were labeled with “T”. Strains obtained from this study are in red. This dataset is made available under the Open Database License (http://opendatacommons.org/licenses/odbl/1.0/). The Open Database License (ODbL) is a license agreement intended to allow users to freely share, modify, and use this Dataset while maintaining this same freedom for others, provided that the original source and author(s) are credited.Junmin Liang, Guangshuo Li, Shiyue Zhou, Meiqi Zhao, Lei Cai

Supplementary material 3 Figure S3. The ML consensus tree inferred based on *cmdA* partial sequence with bootstrap values for ML (> 70%) and posterior probability (PP) (PP > 0.95) labeled to the left of a node (ML/PP) Data type: phylogenetic data Explanation note: The type strains were labeled with “T”. Strains obtained from this study are in red. This dataset is made available under the Open Database License (http://opendatacommons.org/licenses/odbl/1.0/). The Open Database License (ODbL) is a license agreement intended to allow users to freely share, modify, and use this Dataset while maintaining this same freedom for others, provided that the original source and author(s) are credited.Junmin Liang, Guangshuo Li, Shiyue Zhou, Meiqi Zhao, Lei Cai

Supplementary material 4 Figure S4. The ML consensus tree inferred based on *rpb2* partial sequence with bootstrap values for ML (> 70%) and posterior probability (PP) (PP > 0.95) labeled to the left of a node (ML/PP) Data type: phylogenetic data Explanation note: The type strains were labeled with “T”. Strains obtained from this study are in red. This dataset is made available under the Open Database License (http://opendatacommons.org/licenses/odbl/1.0/). The Open Database License (ODbL) is a license agreement intended to allow users to freely share, modify, and use this Dataset while maintaining this same freedom for others, provided that the original source and author(s) are credited.Junmin Liang, Guangshuo Li, Shiyue Zhou, Meiqi Zhao, Lei Cai

Supplementary material 5 Figure S5. The ML consensus tree inferred based on LSU and *rpb2* partial sequences with bootstrap values for ML (> 70%) and posterior probability (PP) (PP > 0.95) labeled to the left of a node (ML/PP) Data type: phylogenetic data Explanation note: The type strains were labeled with “T”. Strains obtained from this study are in red. This dataset is made available under the Open Database License (http://opendatacommons.org/licenses/odbl/1.0/). The Open Database License (ODbL) is a license agreement intended to allow users to freely share, modify, and use this Dataset while maintaining this same freedom for others, provided that the original source and author(s) are credited.Junmin Liang, Guangshuo Li, Shiyue Zhou, Meiqi Zhao, Lei Cai

Supplementary material 6 Table S1. NCBI GenBank accessions of *28S* ribosomal DNA large-subunit sequences (LSU) used in the phylogenetic analyses Data type: phylogenetic data This dataset is made available under the Open Database License (http://opendatacommons.org/licenses/odbl/1.0/). The Open Database License (ODbL) is a license agreement intended to allow users to freely share, modify, and use this Dataset while maintaining this same freedom for others, provided that the original source and author(s) are credited.Junmin Liang, Guangshuo Li, Shiyue Zhou, Meiqi Zhao, Lei Cai
