# Supplementary figures and images for: Myrothecium-like new species from turfgrasses and associated rhizosphere
Source: MycoKeys. 2019 Apr 18;51:29–53. doi: 10.3897/mycokeys.51.31957 (PMC6583245; doi:10.3897/mycokeys.51.31957)

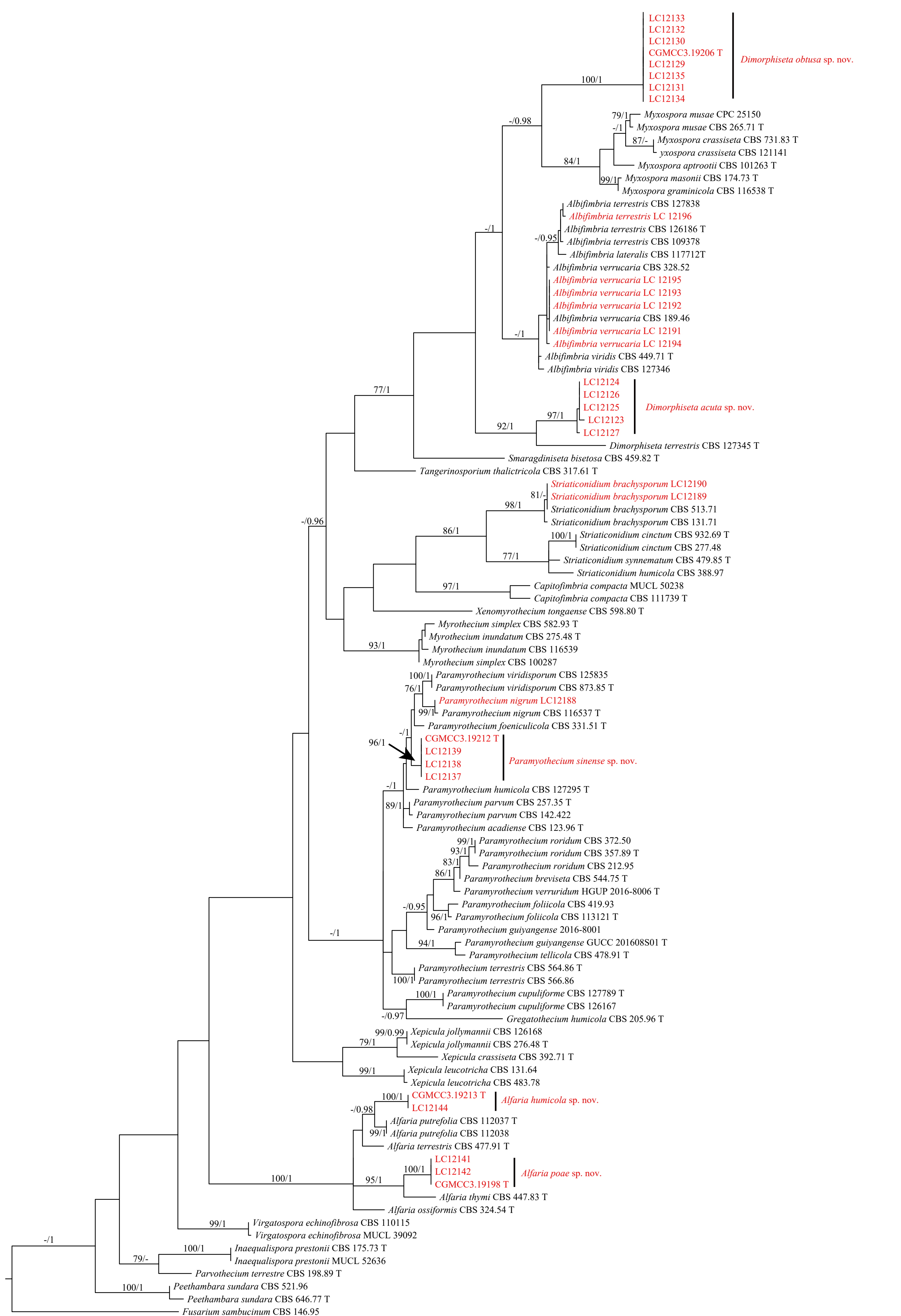

Supplement: Supplementary material 1 [file mycokeys-51-029-s001.pdf]

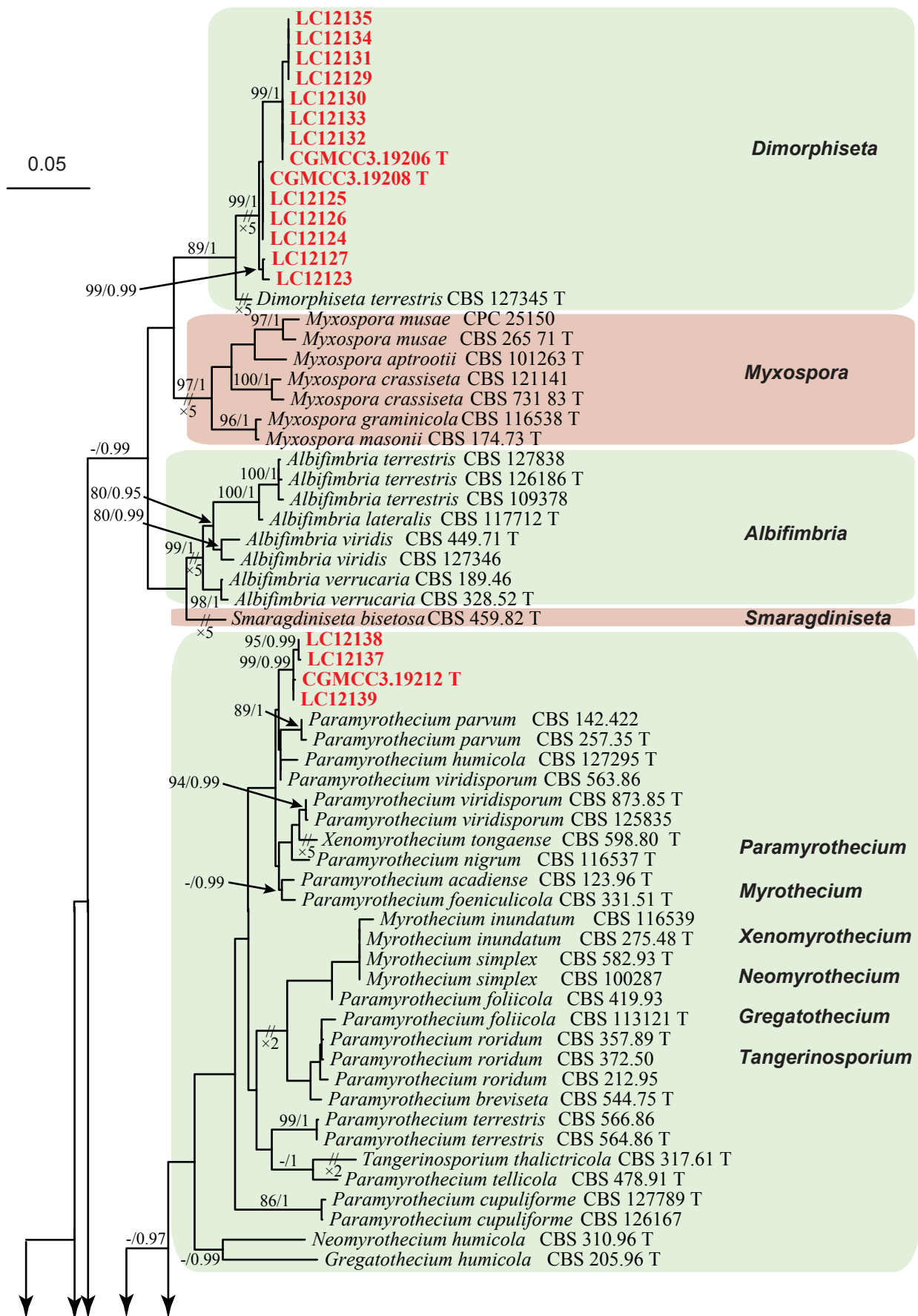

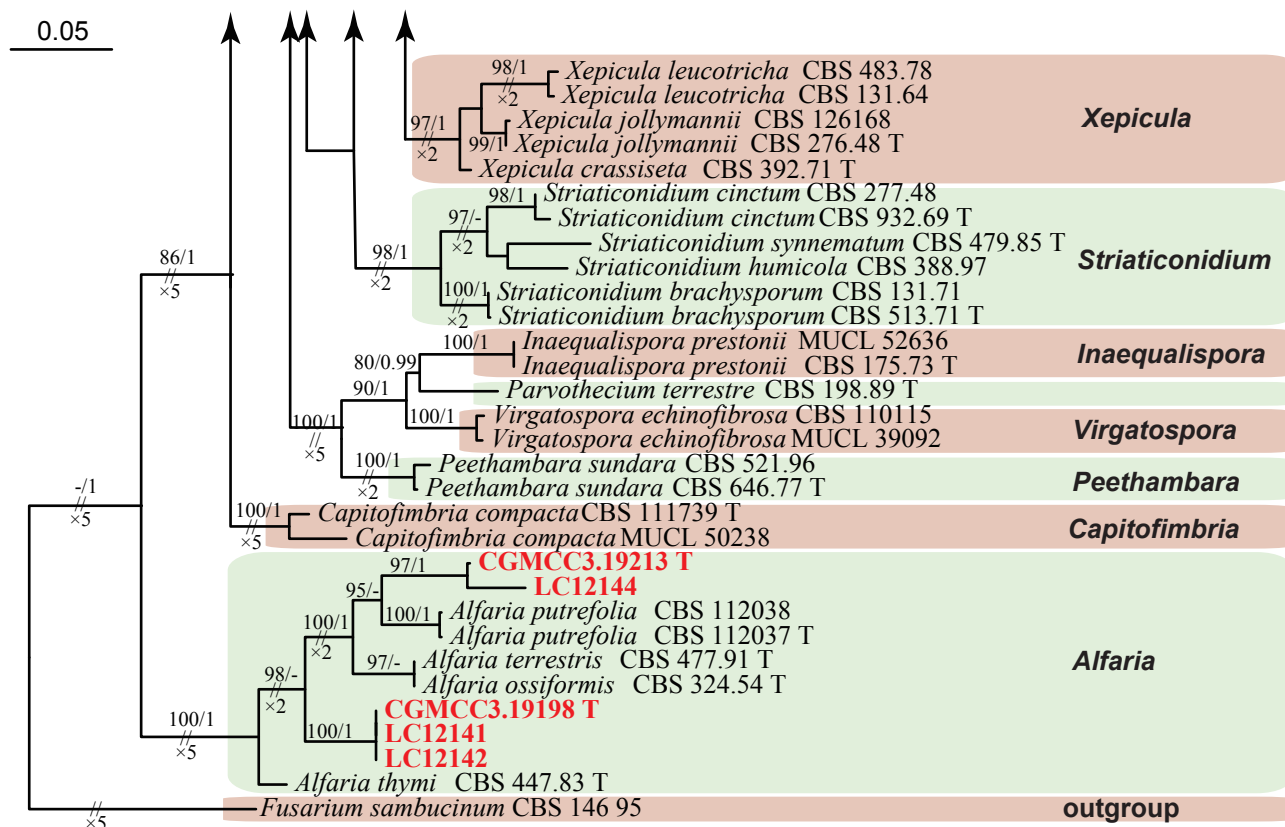

Supplement: Supplementary material 3 [file mycokeys-51-029-s003.pdf]

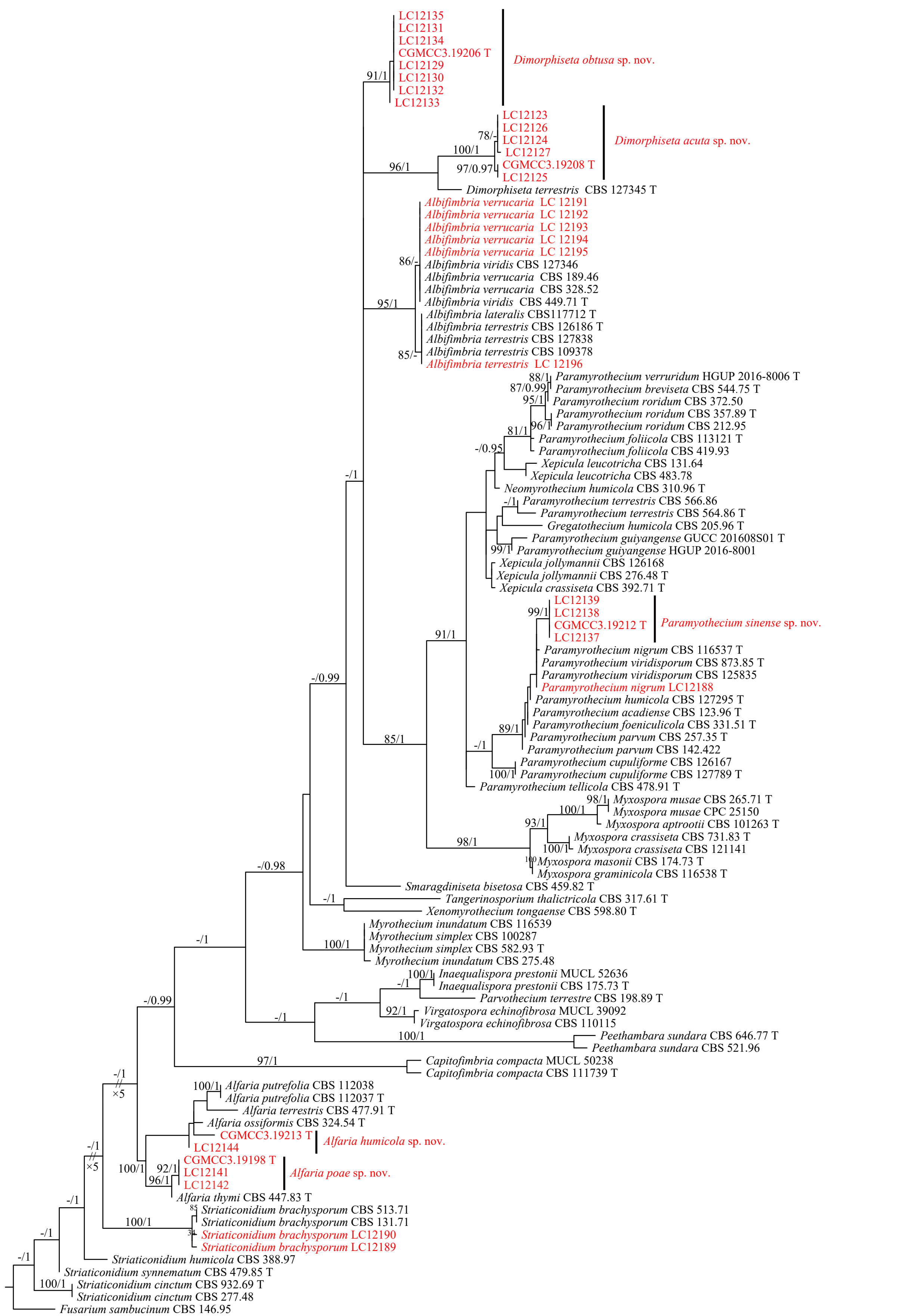

Supplement: Supplementary material 4 [file mycokeys-51-029-s004.pdf]

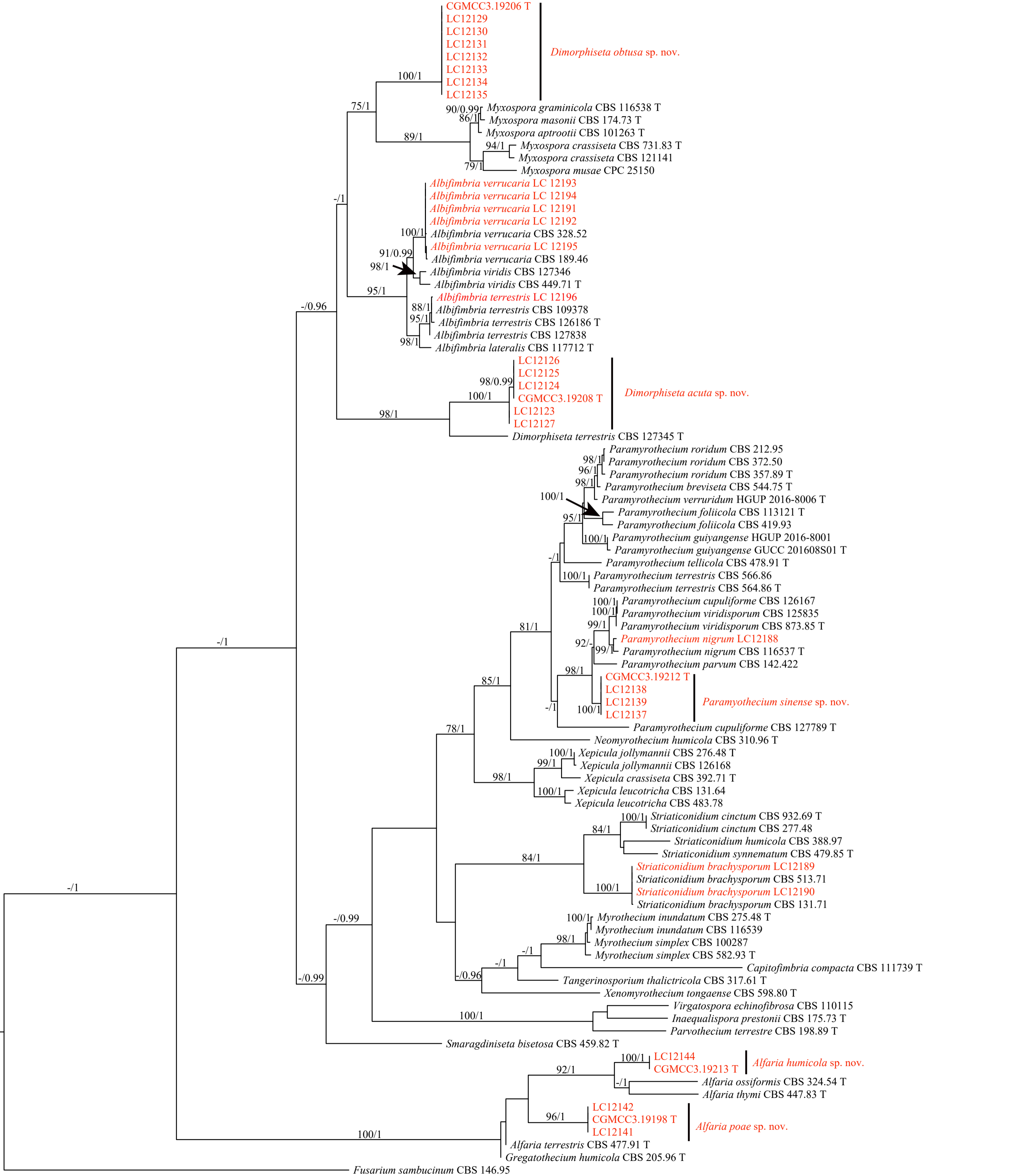

Supplement: Supplementary material 5 [file mycokeys-51-029-s005.pdf]
