# Supplementary material for: Myrothecium-like new species from turfgrasses and associated rhizosphere
Source: MycoKeys. 2019 Apr 18;51:29–53. doi: 10.3897/mycokeys.51.31957 (PMC6583245; doi:10.3897/mycokeys.51.31957)
Supplement: Supplementary material 6 [file mycokeys-51-029-s006.docx]

Table S1 NCBI GenBank accessions of 28S ribosomal DNA large-subunit sequences (LSU) used in the phylogenetic analyses.

| Species | Isolate no. ^a^ | NCBI accession numbers |
| --- | --- | --- |
|  |  | LSU |
| *Myrothecium simplex* | CBS 582.93^T^ | KU846478 |
|  | CBS 100287 | KU846479 |
| *Myr. inundatum* | CBS 275.48^T^  = IMI158855 | KU846474 |
|  | CBS 116539 | KU846476 |
| *Albifimbria lateralis* | CBS117712^T^ | KU845900 |
| *Al. terrestris* | CBS 126186^T^ | KU845902 |
| *Al. terrestris* | CBS 109378  = NRRL 31066 | KU845901 |
| *Al. terrestris* | CBS 127838 | KU845903 |
| *Al. verrucaria* | CBS 328.52^T^  = NRRL 2003  = ATCC 9095 | KU845912 |
| *Al. verrucaria* | CBS 189.46  = IMI 140060 | KU845908 |
| *Al. viridis* | CBS 449.71^T^  = BCC 37540 | KU845917 |
| *Al. viridis* | CBS 127346 | KU845918 |
| *Alfaria. ossiformis* | CBS 324.54^T^ | KU845993 |
| ***Alf. humicola* sp. nov.** | CGMCC3.19213^T^  = LC12143 |  |
|  | LC12144 |  |
| ***Alf. poae* sp. nov.** | CGMCC3.19198^T^  = LC12140 |  |
|  | LC12141 |  |
|  | LC12142 |  |
| *Alf. putrefolia* | CBS 112037^T^ | KU845994 |
|  | CBS 112038 | KU845995 |
| *Alf. terrestris* | CBS 477.91^T^ | KU845997 |
| *Alf. thymi* | CBS 447.83^T^ | KU845999 |
| *Capitofimbria compacta* | CBS 111739^T^ | KU846317 |
|  | MUCL 50238 | KU878557 |
| *Dimorphiseta terrestris* | CBS 127345^T^ | KU846346 |
| ***D. acuta* sp. nov.** | CGMCC3.19208^T^  = LC12122 |  |
|  | LC12123 |  |
|  | LC12124 |  |
|  | LC12125 |  |
|  | LC12126 |  |
|  | LC12127 |  |
| ***D. obtusa* sp. nov.** | CGMCC3.19206^T^  = LC12128 |  |
|  | LC12129 |  |
|  | LC12130 |  |
|  | LC12131 |  |
|  | LC12132 |  |
|  | LC12133 |  |
|  | LC12134 |  |
|  | LC12135 |  |
| *Gregatothecium humicola* | CBS 205.96^T^ | KU846347 |
| *Inaequalispora prestonii* | CBS 175.73^T^ | KU846348 |
|  | MUCL 52636 | KY389337 |
| *Myxospora masonii* | CBS 174.73^T^ | KU846484 |
| *Myx. graminicola* | CBS 116538^T^ | KU846483 |
| *Myx. aptrootii* | CBS 101263^T^ | KU846480 |
| *Myx. musae* | CBS 265.71^T^ | KU846485 |
|  | CPC 25150 | — |
| *Myx. crassiseta* | CBS 731.83^T^ | KU846481 |
|  | CBS 121141  = NRRL 45891 | KU846482 |
| *Neomyrothecium humicola* | CBS 310.96^T^ | KU846488 |
| *Peethambara sundara* | CBS 646.77^T^ | AF193245 |
|  | CBS 521.96  = MUCL 39093 | KU846491 |
| *Parvothecium terrestre* | CBS 198.89^T^ | KU846489 |
|  | CBS 534.88  = INIFAT C87/234 | KU846490 |
| *Paramyrothecium humicola* | CBS 127295^T^ | KU846325 |
| *P. parvum* | CBS 257.35^T^ | KU846328 |
|  | CBS 142.422  = IMI 155923  = MUCL 7582 | KU846327 |
| *P. foeniculicola* | CBS 331.51^T^ | KU846322 |
| *P. nigrum* | CBS 116537^T^ | KU846326 |
| *P. cupuliforme* | CBS 127789^T^ | KU846321 |
| *P. viridisporum* | CBS 873.85^T^ | KU846338 |
|  | CBS 125835 | KU846340 |
| *P. acadiense* | CBS 123.96^T^ | KU846318 |
| *P. terrestris* | CBS 564.86^T^ | KU846333 |
|  | CBS 566.86 | KU846335 |
| *P. tellicola* | CBS 478.91^T^ | KU846332 |
| *P. foliicola* | CBS 113121^T^  = INIFAT C02/104 | KU846324 |
|  | CBS 419.93  = INIFAT C93/60 | KU846323 |
| *P. breviseta* | CBS 544.75^T^ | KU846319 |
| *P. roridum* | CBS 357.89^T^ | KU846330 |
|  | CBS 212.95 | KU846329 |
|  | CBS 372.50  = IMI 140050 | KU846331 |
| ***P. sinense* sp. nov.** | CGMCC3.19212^T^ = LC12136 |  |
|  | LC12137 |  |
|  | LC12138 |  |
|  | LC12139 |  |
| *Smaragdiniseta bisetosa* | CBS 459.82^T^ | KU847255 |
| *Striaticonidium brachysporum* | CBS 513.71  = IMI 115293^T^ | KU847258 |
| *Str. brachysporum* | CBS 131.71  = IMI 158441  = ATCC 22270 | KU847256 |
| *Str.synnematum* | CBS 479.85^T^ | KU847268 |
| *Str. cinctum* | CBS 932.69^T^ | KU847265 |
|  | CBS 277.48  = IMI 001526 | KU847262 |
| *Str. humicola* | CBS 388.97 | KU847267 |
| *Tangerinosporium thalictricola* | CBS 317.61^T^  = IMI 034815 | KU847269 |
| *Virgatospora echinofibrosa* | CBS 110115 | KU847270 |
|  | MUCL 39092  = ATCC 200437 | KU847271 |
| *Xenomyrothecium tongaense* | CBS 598.80^T^ | KU847272 |
| *Xepicula crassiseta* | CBS 392.71^T^ | KU847273 |
| *X. jollymannii* | CBS 276.48^T^  = MUCL 11830 | KU847274 |
|  | CBS 126168 | KU847276 |
| *X. leucotricha* | CBS 131.64  = IMI 103664  = ATCC 16686 | KU847277 |
|  | CBS 483.78 | KU847280 |
| *Fusarium sambucinum* | CBS 146.95 | KM231682 |

^a^ BCC: BIOTEC Culture Collection, National Center for Genetic Engineering and Biotechnology (BIOTEC), Bangkok, Thailand; CBS: CBS-KNAW Fungal Diversity Centre, Utrecht, The Netherlands; CGMCC: China General Microbioglogical Culture Collection Center,Beijing; CPC: Collection of P.W. Crous; MUCL: Mycothèque de l’Université Catholique de Louvian, Belgium.

^T^ Ex-type and ex-epitype cultures
